# *Vaccinia virus* in Feces and Urine of Wild Rodents from São Paulo State, Brazil

**DOI:** 10.3390/v10020051

**Published:** 2018-01-23

**Authors:** Marina G. Peres, Thais S. Bacchiega, Camila M. Appolinário, Acácia F. Vicente, Mateus S. R. Mioni, Bruna L. D. Ribeiro, Clóvis R. S. Fonseca, Vanessa C. Pelícia, Fernando Ferreira, Jonatas S. Abrahão, Jane Megid

**Affiliations:** 1 Faculdade de Medicina Veterinária e Zootecnia, UNESP—Universidade Estadual Paulista, Botucatu CEP 18618-970, Brazil; marinageavet@yahoo.com.br (M.G.P.); tatabacch@hotmail.com (T.S.B.); camilaapp.vet@gmail.com (C.M.A.); acaciavicente@hotmail.com (A.F.V.); mateusmioni@yahoo.com.br (M.S.R.M.); brunadevider@gmail.com (B.L.D.R.); crsfonseca2@yahoo.com.br (C.R.S.F.); vcpelicia@yahoo.com.br (V.C.P.); 2 Faculdade de Medicina Veterinária e Zootecnia, USP—Universidade de São Paulo, São Paulo CEP 05508-270, Brazil; fernando@vps.fmvz.usp.br; 3 Instituto de Ciências Biológicas, UFMG—Universidade Federal de Minas Gerais, Belo Horizonte CEP 31270-901, Brazil; jonatas.abrahao@gmail.com

**Keywords:** *Vaccinia virus*, epidemiology, transmission, public health, wild animals, PCR

## Abstract

The origin of *Vaccinia virus* (VACV) outbreaks in Brazil remains unknown, but since the isolation of VACV in *Mus musculus* mice during a zoonotic outbreak affecting cattle and milkers, peridomestic rodents have been suggested to be a link between cows and wild animals. Considering that experimentally infected mice eliminate viral particles in their feces, we investigated the presence of VACV in the feces and urine of wild rodents that were captured in the forest areas surrounding milking farms in the central west region of São Paulo State. For the first time, this work reports the detection of VACV by PCR in the feces of naturally infected *Oligoryzomys flavescens, Oligoryzomys nigripes,* and *Sooretamys angouya,* and in the urine of *Oligorizomys flavescens,* which raises important questions about the spread of VACV by rodent feces and its potential to induce clinical infections in cows.

## 1. Introduction

*Vaccinia virus* (VACV) is a prototype of the *Orthopoxvirus* genus (OPV) that has been the causal agent of emergent exanthematous zoonotic outbreaks in Brazil over the past decade [1,2,3,4]. The emergence of human cases of VACV infection may be related to the end of the intensive vaccination campaign against smallpox promoted by the World Health Organization (WHO) in 1980, but the origin of the outbreak in cattle remains unknown [5,6].

A hypothetical transmission model has been suggested following the isolation of VACV from *Mus musculus* mice during a zoonotic outbreak [7]. In this model, peridomestic rodents act as a connection between wildlife and domestic animals in rural areas [7]. In Europe, wild and peridomestic rodents are known to act as reservoirs of cowpox [3], but in Brazil, the role of these rodents as VACV reservoirs remains unclear. Although mice that are experimentally infected eliminate viable viral particles in their feces [8,9], transmission to cows through contact with rodent feces has not been established during any of the Brazilian outbreaks.

In the present study, we assessed the presence of VACV in fecal and urine samples from wild rodents captured in forest areas surrounding milking farms in areas with and without histories of VACV zoonotic outbreaks.

## 2. Materials and Methods

This study was approved by Ethical Committee of Animals Uses in Veterinary Medicine and Animal Production UNESP, Botucatu, São Paulo (number 114/2015-CEUA, 10/03/2015), the Brazilian Institute of Renewable Environment and Natural Resources (IBAMA) of the Environment Ministry (MMA), the Chico Mendes Biodiversity Conservation Institute (ICMBio), and the Biodiversity Information and Authorization System (SISBIO) for wild rodent capture under the number 23918-1.

**Figure 1:**
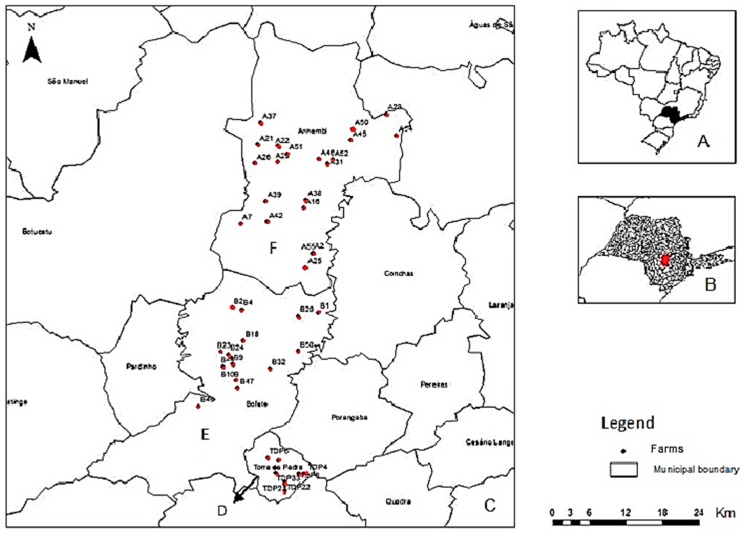
Map of the sampling sites in Brazil (**A**) with São Paulo State shown in black. São Paulo state map (**B**) with Torre de Pedra, Bofete, and Anhembi in red. Map of São Paulo State (**C**) showing the sampling sites; the points in red correspond to the farms in Torre de Pedra (**D**); Bofete (**E**); and Anhembi (**F**). Source: Peres et al., 2013 [14].

Samples were collected in three counties in the central west region of São Paulo State with and without histories of VACV zoonotic outbreaks, i.e., Torre de Pedra (23°14′58.76″ S, 48°11′39.49″ W), in which outbreaks were registered in 2007 and 2010 [10,11] and Bofete (23°05′54.51″ S, 48°11′26.61″ W) and Anhembi (23°05′54.51″ S, 48°11′26.61″ W), in which histories of outbreaks are unknown (Figure 1).

The total number of farms included in the study was calculated based on the populations of the farms in the three counties, a 5% prevalence of positive farms (at least one positive sample), and a 5% margin of error using Epi Info 3.5.4. Forty-seven farms were randomly selected, including 10 in Torre de Pedra, 15 in Bofete, and 22 in Anhembi (Figure 1).

Wild rodents were captured from May to September of 2011 on 47 milking farms in the three counties (Figure 1). Pitfall and Sherman traps were used for wild rodent capture, and peanut cream, canned sardines, cornmeal, and oatmeal were used as bait. Five trap nights were required to capture animals in each of the native forest areas (consisting of a transitional Atlantic Forest and Cerrado) surrounding each milking farm. Due to the risk of exposure to infectious diseases related to rodents, such as *Hantavirus*, all procedures were performed following safe procedures. Positive pressure masks with High Efficiency Particulate Arrestance (HEPA) filters and triple glove layers were used during the procedures for checking the Sherman and Pitfall traps. Rodents in the Pitfall traps were removed and placed in plastic boxes for transport to the site of sample collection, and the Sherman traps containing rodents were transported to the site of sample collection.

The personal protective equipment (PPE) consisted of a waterproof polypropylene disposable apron, two pairs of procedure gloves, rubber boots, and a motorized respiratory set containing a Tyvex-type cap, a trachea, and a motor; additionally, a HEPA filter was used during the collection of the wild rodent samples. The rodents were anesthetized in plastic autoclavable bags containing gauze soaked in ethyl ether. Blood samples were collected by cardiac puncture, and if death did not occur after the puncture, they were euthanized through the deepening of the anesthetic plan. The organs, feces, and urine were collected (feces directly from the final portion of the intestine, and urine by puncturing the urinary bladder with insulin needle), placed into microtubes, and stored at −80 °C until the polymerase chain reaction (PCR) assays.

The PPE described for the wild rodent sample collection was also used during the viral DNA extraction from the feces and urine samples, and these steps were performed in a laminar flow hood.

Viral DNA was extracted using the RTP^®^ DNA/RNA Virus Mini Kit (Stratec Molecular, Berlin, Germany) with ultrapure water as a negative control every 3 samples. A nested PCR was used for the amplification of the vaccinia growth factor (*vgf*) gene [12]. The *vgf* gene is a conserved *Orthopoxvirus* (OPV) gene, widely used as a PCR target, in diagnostic and phylogenetic Brazilian VACV outbreaks [12,13]. The nested PCR was carried out in a two-step reaction protocol. In the first step, were used the OPV primers (vgfF: CGCTGCTATGATAATCAGATCATT and vgfR: GATATGGTTGTGCCATAATTTTTAT). In the nested step, the pair of internal OPV primers (vgfF2: ACACGGTGACTGTATCCA and vgfR2: CTAATACAAGCATAATAC) were used [12]. In the first step, 2 μL of template were added to 18 μL of the PCR reaction mixture containing 0.4 mM of OPV primers (VGF-F and VGF-R), 10mM dNTPs, 2.0 mM MgCl_2_, 500 ng Bovine Serum Albumin (BSA), and 2 U of Taq DNA polymerase, using the manufacturer’s supplied 10× buffer. Reactions were performed using the following protocol: incubation at 95 °C for 9 min; 30 cycles of denaturation (94 °C, 1 min), annealing (45 °C, 1min), extension (72 °C, 1 min), and final extension (72 °C, 10 min). The nested PCR step was carried out using 1 μL of undiluted first PCR product as template. The same chemical and thermal conditions were used, but using internal OPV primers (vgfF2 and vgfR2). PCR sensitivity of this was determined with decimal serial dilutions ranging from 10^4^ to 1 PFU of VACV-WR as templates, and the sensitivity was defined by the highest viral dilution detected by PCR [13]. Positive samples were submitted to gene sequencing, and a phylogenetic tree was constructed using the neighbor-joining method; the Tamura-3 model of nucleotide substitutions and a bootstrap of 1000 replicates in MEGA 7.0 (Pennsylvania State University, State College, PA, USA).

## 3. Results

### 3.1. Wild Rodent Capture and Sample Collection

A total of 138 wild rodents of the following species were captured: Akodon montensis, Calomys tener, Juliomys pictipes, Necromys lasiurus, Nectomys squamipes, Oligoryzomys flavescens, Oligoryzomys nigripes, and Sooretamys angouya (Table 1).

Fecal samples were collected from 115 animals, and urine samples were also collected from 55 animals (Table 1). For some animals, fecal and/or urine sample collection was not possible due to the absence of intestinal content and/or empty urinary bladders. The most sampled species was *Oligoryzomys nigripes* (54%), following by *Oligoryzomys flavescens* (22%) and *Sooretamys angouya* (10%) (Table 1). The numbers of analyzed fecal and urine samples were also highest for these species (Table 1).

### 3.2. Polymerase Chain Reaction (PCR) and Sequencing

Among the 115 analyzed fecal samples, six (5.2%) were positive for vaccinia growth factor (*vgf*) gene amplification by nested PCR, including three samples from Anhembi (4%) and three from Bofete (8%); however, only one of the positive samples was sequenced due to the fact that the low DNA concentration of the other five were not sufficient for sequencing (Table 2). The percentages of positivity of the fecal samples from the different species were 14% for *Sooretamys anoguya*, 8% for *Oligoryzomys flavescens*, and 4% for *Oligoryzomys nigripes*. According to county, the highest rates of positivity were observed in *Oligoryzomys flavescens* from Bofete (22%) and *Sooretamys angouya* from Anhembi (15%) (Table 2).

Among the 55 analyzed urine samples, only one (1.8%) was positive and was not sequenced due to the low DNA concentration (Table 3). The only species with a positive urine sample was *Oligoryzomys flavescens* (9%) from Anhembi (14%). All wild rodents sampled in the present study had been previously tested for detection of neutralizing antibodies against *Orthopoxvirus* [14]. The simultaneous detection of VACV DNA in the feces and neutralizing antibodies against *Orthopoxvirus* (OPV) was observed in two *Oligoryzomys flavescens* from Bofete (Table 3).

### 3.3. Phylogenetic Analysis

**Figure 2:**
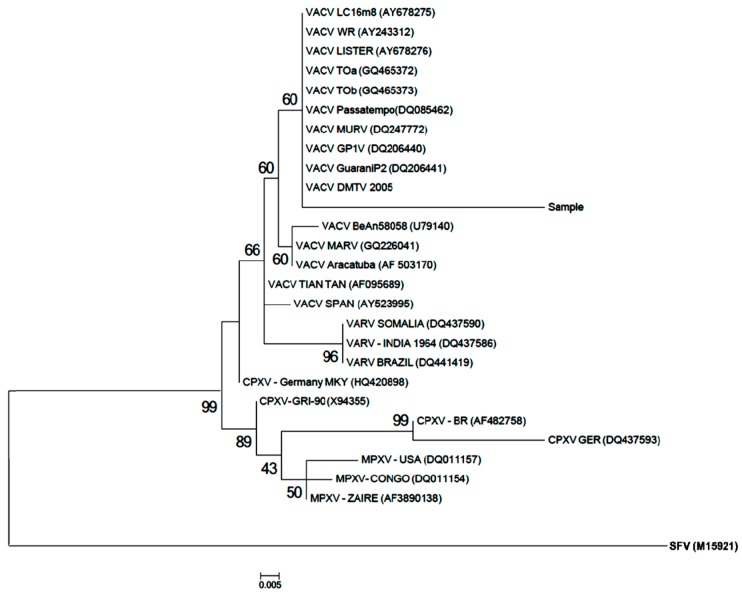
Phylogenetic tree based on the OPV nucleotide sequence of the *vgf* gene showing the rodent strain (sample) cluster. In bold, the out group shope fibroma virus (SFV).

**Figure 3:**
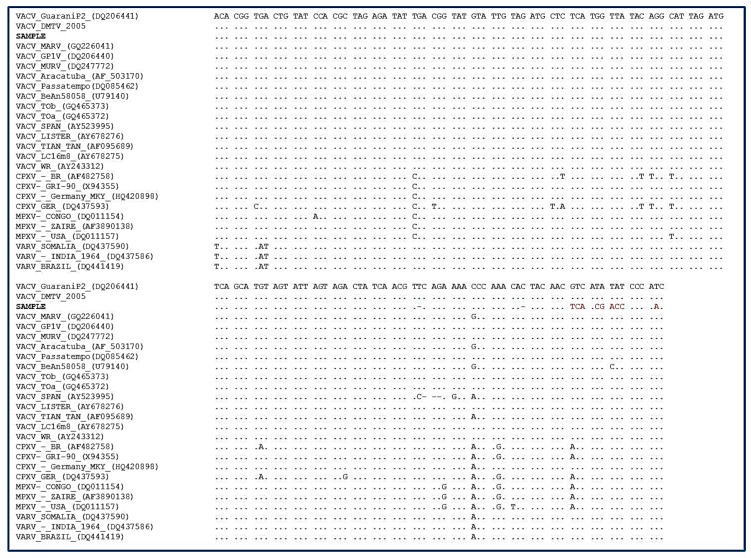
Alignment of the rodent strain (SAMPLE) sequences showing the polymorphism in red.

The sequencing and phylogenetic tree based on the orthopoxvirus nucleotide sequence of the *vgf* gene revealed that our strain (sample) clustered with the Brazilian VACVs (i.e., TOa, TOb, Passatempo, MURV, GP1V, GP2V, and DMTV2005) and vaccine VACVs (i.e., Lc16m2, WR, and Lister), which characterized it as a *Vaccinia virus*. (Figure 2); however, the *vgf* gene exhibited nucleotide polymorphisms that indicated that it was not completely similarly to the clustered VACV (Figure 3). The mutations observed at the 3`region of virus growth factor gene of our isolate (Figure 3, red highlighted) resulted in a change in predicted amino acids sequences, from SQNPNTTTSYIP to SQNPNLQLNDPQ.

## 4. Discussion

In previous work, we reported low seropositivity to orthopoxviruses among wild rodents sampled in in the southwest region of the state of São Paulo with or without official reports of VACV outbreaks in cattle or humans, which leads us to believe that *Oligoryzomis nigripes*, *Oligoryzomis flavenscens*, and *Sooretamys angouya* were not VACV reservoirs in this Brazilian region [14]. Conversely, in this paper we report for the first time the detection of VACV by PCR in the feces and urine in these naturally infected animals captured in forest areas surrounding milk farms from this area and previously evaluated for seropositivity to VACV. These findings agree with the results of previous studies in which mice that were experimentally infected with VACV were able to eliminate viral particles in their feces and urine [8].

The presences of VACV in 5.2% of the fecal samples and 1.8% of the urine samples analyzed are low and corroborate previous serologic findings that demonstrated 8.7% seropositivity in the same animals studied [14]. Only two positive wild rodents (*Oligoryzomys flavescens*) have previously exhibited the presence of antibodies against orthopoxviruses. The absence of positive wild rodents in Torre de Pedra, which is a county with a history of official reports of VACV zoonotic outbreaks [10,11], is also in accordance with the absence of seropositivity in wild rodents from this region [14]. In contrast, Anhembi and Bofete, both cities without histories of VACV zoonotic outbreak, presented positive PCR results, and these findings accord with previous seropositivities observed in wild rodents sampled from these counties [14].

These results allow us to question the epidemiological influence of viral shedding in the feces of wild rodents in terms of the transmission of VACV to cattle. Long-lasting stabilities of VACV strains in the feces of experimentally infected BALB/c mice have been demonstrated [9], and the transmission of VACV via the feces of experimentally infected cows to mice has previously been described [15].

However, transmission from the feces of experimentally infected mice has been described only for exposed sentinel BALB/c mice [8]. A peridomestic rodent (*Mus musculus*) was once found to be positive for VACV during a zoonotic outbreak [7], but no wild rodent species have been positively diagnosed during VACV outbreaks. *Mus musculus* is an Old World species that was introduced to Brazil [16]. In Europe, among other peridomestic species, such as *Rattus rattus* and *Rattus norvergicus*, this species acts as a natural reservoir of *Cowpox virus* (CPXV) [3,17]. In recent years, cases of CPXV transmission from pet rats (*Rattus norvergicus*) to pet owners, from rats to other pet animals, and from these pets to pet owners have been described. Nevertheless, in these cases, the involved animals all became sick and died, and transmission occurred via direct contact between humans and the animals’ lesions [18,19]. Indeed, the transmission of VACV via the feces of experimentally infected mice to cows has not yet been described, and no cases of rodent-to-cow or rodent-to-human VACV transmission have been reported during the Brazilian outbreaks.

Another relevant finding from an investigation of the spread of VACV via murine feces is that sentinel BALB/c mice that are naturally infected by direct contact with the feces of experimentally infected mice do not exhibit any clinical signs independent of whether group I or group II VACVs are examined, which suggests that these mice experience subclinical infections [8]. Similar observations have been made with BALB/c mice that have been exposed to the feces of experimentally infected cows [15]. Taking these findings into account, along with the facts that both VACV and CPXV are transmitted by direct contact with lesions, questions are raised concerning whether the low viral loads eliminated in the feces are able to infect and produce disease in cattle.

We use the amplification of *vgf* gene to screen for positive wild rodents due to high sensitivity of this PCR reaction [12,13]. Among the six positive samples, the DNA concentration of one was of sufficient quality for sequencing, whereas the DNA concentration of the other five was not. We ascribe the low DNA concentration to low viral loads in the feces once the PCR method used had the sensitivity of 10^2^–10^3^ PFU for feces and urine, respectively [13]. Therefore, it is quite likely that these animals were in a subclinical infection stage, because they did not exhibit clinical signs, and the viral loads eliminated in their feces were low. Thus, these animals may not be capable of transmitting sufficient viral loads to induce clinical infection in cattle considering that previous experiments used 50 µL of 10^6^ PFU/mL for successful experimental infection [20]. Although not mentioned in the materials and methods, we attempted to classify the VACV in one of two groups of Brazilian VACVs according to the deletion of amino acids A56R in the gene that encodes the viral hemagglutinin (*ha* gene). Thus, all samples were also tested for the amplification of the *ha* gene according to the methods of Damaso et al. [21], but these tests were not successful. This protocol is less sensitive and does not detect low viral loads, which reinforces our hypothesis that the viral loads eliminated in the sampled feces were low.

Interestingly, during a zoonotic outbreak in São Paulo State, three dogs and three opossums (*Didelphis albiventris*) without characteristic clinical signs were found to be positive for VACV by PCR among all of the tested blood samples, but no positive wild rodent (*Akodon montensis* and *Nectomys squamipes*) samples were found [22]. These findings are similar to those of a previous serologic study in which a high prevalence of antibodies against *Orthopoxvirus* (OPV) was detected in dogs without clinical signs, which suggests their involvement in the spread of VACV [14]. Other wild species, such as capuchin monkeys (*Cebus paella*) and howler monkeys (*Alouata carya*), have been found to be positive in serological and molecular tests without presenting clinical signs [23]. Thus, the involvement of rodents in the spread of VACV and the involvements of other mammalian species, both domestic and wild, require further study.

Our study detected VACV DNA in wild rodent feces and raises important questions about the spread of *Vaccinia virus* by rodent feces and its potential to induce clinical infections in cows. Additional studies are needed to further elucidate this epidemiologic situation.

## Figures and Tables

**Table 1:** Wild rodent species captured and clinical samples analyzed for presence of VACV in Brazil.

Species	Captured		Analyzed Samples			
			Feces		Urine	
	*n*	(%) *	*n*	(%) *	*n*	(%) *
*J. pictipes*	1	(0.7)	0	(0.0)	0	(0.0)
*N. lasiurus*	1	(0.7)	1	(0.9)	1	(1.8)
*C. tener*	4	(2.9)	4	(3.5)	1	(1.8)
*N. squamipes*	4	(2.9)	3	(2.6)	2	(3.6)
*A. montensis*	8	(5.8)	6	(5.2)	2	(3.6)
*S. agouya*	14	(10.1)	14	(12.2)	9	(16.4)
*O. flavescens*	31	(22.5)	24	(20.9)	11	(20.0)
*O. nigripes*	75	(54.3)	63	(54.8)	29	(52.7)
TOTAL	138	(100.0)	115	(83.3)	55	(39.8)

* Percentages calculated over the total numbers of samples.

**Table 2:** Distributions of wild rodent fecal samples according to municipality and VACV positivity in Brazil.

Species	Anhembi		Bofete		Torre de Pedra		TOTAL	
	*n*	Positive (%)	*n*	Positive (%)	*n*	Positive (%)	*n*	Positive (%)
*N. lasiurus*	0	0 (0.0)	1	0 (0.0)	0	0 (0.0)	1	0 (0.0)
*N. squamipes*	2	0 (0.0)	1	0 (0.0)	0	0 (0.0)	3	0 (0.0)
*C. tener*	2	0 (0.0)	2	0 (0.0)	0	0 (0.0)	4	0 (0.0)
*A. montensis*	0	0 (0.0)	6	0 (0.0)	0	0 (0.0)	6	0 (0.0)
*S. agouya*	13	2 (15.4)	1	0 (0.0)	0	0 (0.0)	14	2 (14.3)
*O. flavescens*	14	0 (0.0)	9	2 (22.2)	1	0 (0.0)	24	2 (8.3)
*O. nigripes*	42	1 * (2.3)	18	1 (5.5)	3	0(0.0)	63	2 (3.7)
TOTAL	73	3 (4.1)	38	3 (7.9)	4	0 (0.0)	115	6 (5.2)

* Sequenced sample.

**Table 3:** Correlation between detection of VACV DNA in fecal samples and previous seropositivity (SN) in the same sampled wild rodents [14].

Species	Anhembi			Bofete			Torre de Pedra			TOTAL		
	*n*	Positive		*n*	Positive		*n*	Positive		*n*	Positive	
		DNA	SN ^1^		DNA	SN ^1^		DNA	SN ^1^		DNA	SN ^1^
*N. lasiurus*	0	0	0	1	0	0	0	0	0	1	0	0
*N. squamipes*	2	0	0	1	0	0	0	0	0	3	0	0
*C. tener*	2	0	0	2	0	0	0	0	0	4	0	0
*A. montensis*	0	0	0	6	0	0	0	0	0	6	0	0
*S. agouya*	13	2	2	1	0	0	0	0	0	14	2	2
*O. flavescens*	14	0	1	9	2 *	2 *	1	0	0	24	2	3
*O. nigripes*	42	1	2	18	1	2	3	0	0	63	2	4
TOTAL	73	3	5	38	3	4	4	0	0	115	6	9

DNA = positive fecal samples for detection of VACV DNA by PCR; SN = positive serum samples for detection of Neutralizing Antibodies by Plaque Reduction Neutralizing Test (PRNT) previously observed in the same sampled wild rodents [14]; ^1^ data from previous serologic study [14]; * wild rodents, including positive, PCR, and PRNT.
